# Natriuretic peptides, via GC-A/cGMP, moderate hypoxia-induced VEGF release from astrocytes and thereby pathological neovascularization in the retina

**DOI:** 10.1186/2050-6511-16-S1-A89

**Published:** 2015-09-02

**Authors:** Katarina Spiranec, Sabrina Hupp, Birgit Gaßner, Aleksandra Sindic, Michaela Kuhn

**Affiliations:** 1 Institute of Physiology, University of Würzburg, Germany; 2 Department of Physiology and Immunology, School of Medicine, University of Zagreb, Croatia

## Background

Our previous studies demonstrated that natriuretic peptides, i.e. BNP produced by activated satellite cells within ischemic skeletal muscle, stimulate the regeneration of neighboring endothelia via endothelial GC-A/cGMP signaling [1]. This paracrine communication might be critically involved in coordinating postischemic muscle regeneration and angiogenesis. In the retina, angiogenesis occurs as a part of normal development as well as in proliferative vascular diseases, such as diabetic retinopathy (DR) or retinopathy of prematurity (ROP) [2]. Retinal vascular development is controlled by interactions between ganglion cells, astrocytes and endothelial cells. In particular, reciprocal feedback between endothelial cells and astrocytes is crucial for proper vascular patterning. Hypoxia-induced vascular endothelial growth factor (VEGF) expression in astrocytes plays a key role in (patho)physiological retinal endothelial growth. Notably, immunohistochemistry on postnatal (P7) retinal whole-mounts revealed the expression of immunoreactive BNP in glial fibrillary acidic protein (GFAP)–expressing astrocytes. Therefore we postulated that BNP participates in this astrocyte-endothelial communication during physiological vascularization and/or pathological revascularization of the retina.

## Materials and methods

We compared physiological postnatal retinal vascularization in mice with conditional, either endothelial (EC GC-A KO [1]) or astrocyte–restricted deletion of GC-A (astrocyte GC-A KO) and respective control littermates (GC-A^fl/fl^). The latter mouse model was generated by crossing GC-A^fl/fl^ mice with GFAP-Cre^TG^ mice. In addition, we studied pathological neoangiogenesis in oxygen-induced retinopathy (OIR), a disease model for DR and ROP [3]. These *in vivo* studies were complemented with electrophysiological and molecular studies (intracellular cGMP, VEGF secretion) in primary cultured murine brain astrocytes.

## Results

Firstly we studied whether crossing GC-A^fl/fl^ mice with GFAP-Cre^TG^ mice resulted in efficient and selective inactivation of GC-A in astrocytes. Indeed, arterial blood pressure and GC-A expression levels in peripheral tissues of GC-A^fl/fl^;GFAP-Cre^+/-^mice were unaltered. ANP and BNP enhanced cGMP levels in cultured control astrocytes and these effects were significantly attenuated in astrocytes prepared from GC-A^fl/fl^;GFAP-Cre^+/-^mice. Since these cultures contained ~10% contaminating cells such as microglia, we also performed single-astrocyte patch-clamp recordings. ANP and BNP provoked membrane depolarizations in control astrocytes by ΔVm 1.58 ± 0.46 mV (at 10 nM NPs) and 2.08 ± 0.51 mV (100 nM NPs). In GC-A^fl/fl^;GFAP-Cre^+/-^astrocytes, NP-induced depolarizations were almost abolished: ΔVm 0.59 ± 0.35 and 0.25 ± 0.4 mV at 10 and 100 nM of the NPs.

Retinal whole-mount stainings with FITC-isolectin demonstrated that physiological vasculogenesis 5 and 7 days after birth was unaltered both in EC GC-A KO and astrocyte GC-A KO mice (compared with respective control littermates). In the OIR model, pathological vascular regression (at P12) was also unaltered in both genotypes. However, unexpectedly, hypoxic neovessel formation (at P17) was unchanged in EC GC-A KO but enhanced in astrocyte GC-A KO mice. GFAP stainings revealed unaltered astrocyte density within and around the neovascular zones of astrocyte GC-A KO retinas. Hence, since astrocyte GC-A disruption apparently does not change the vitality/proliferation of these cells but still enhances the pathological proliferation of adjacent endothelia, we hypothesized that NP/GC-A signaling modulates astrocyte VEGF release.

To follow this hypothesis lastly we studied the effects of ANP and BNP on VEGF secretion by cultured astrocytes. Hypoxia (1% O_2_ during 6 or 24h) increased VEGF secretion by 21 fold. ANP and BNP did not significantly modulate basal VEGF secretion but markedly and concentration-dependently attenuated the stimulation by hypoxia. These inhibitory effects were almost fully abolished in GC-A–deficient astrocytes.

## Conclusions

Our observations indicate that BNP and/or ANP, via GC-A/cGMP signaling, participate in a local, autocrine or paracrine feedback which inhibits hypoxia-induced VEGF release from retinal astrocytes and thereby moderates pathological neoangiogenesis. The modulation of VEGF-mediated communication between astrocytes and endothelial cells by natriuretic peptides may have a key role during processes of pathological neoangiogenesis in the brain and retina.
